# Editorialets

**Published:** 1907-04

**Authors:** 


### Editorialets.

MODERN MEDICAL ETHICS: Strain at a "Proprietary" and swallow an "Osteopath."

THE NEW DISPENSATION "dispenses" with professional pride, the honorable traditions and high ideals of regular medicine, with its Principles of Ethics, and, in fact, with everything the older members have been taught and have taught and practiced and that distinguish legitimate medicine from the fungi — called the various "pathies."

DR. Z. T. BUNDY of Millford, Texas, has been reappointed surgeon to the State Confederate Home at Austin.

THE TEXAS STATE MEDICAL ASSOCIATION will hold its thirty-eighth annual meeting at Mineral Wells, Tuesday, Wednesday and Thursday, May 7th, 8th and 9th, prox.

DR. C. B. COMBES of Brownsville, Texas, died April 1 (inst.) at his home in that city, aged 70. He was one of the landmarks of Southwest Texas. Settling in Brownsville in 1859, he was identified with its every interest in all this time. He served as surgeon in the Confederate Army under General Magruder.

DR. T. O. MAXWELL, for twelve years in the service of the State in its insane asylums, and the late superintendent of the Southwestern Insane Asylum at San Antonio, has accepted the position of medical director of the Tuberculosis Sanitarium at Llano, a private institution, and has taken charge of it. The owners are to be congratulated on securing Dr. Maxwell.

DR. M. M. SMITH of Austin has been appointed medical director or chief medical examiner of the Order of Praetorians of Texas.

BARS UP AT ATLANTIC CITY.—"No drug, chemical or similar preparation used in the treatment of diseases can be exhibited which does not conform to the requirements of the Council on Pharmacy and Chemistry of the American Medical Association. A copy of these requirements enclosed.

"No medical journal or publication can be exhibited that contains advertisements of drug, chemical or similar preparation used in the treatment of disease, which does not conform to the rules of the Council on Pharmacy and Chemistry of the American Medical Association."

Abstract from circular on "Regulations Regarding Exhibits," Atlantic City, N. J., on the occasion of the Fifty-eighth Annual Session of the American Medical Association, June 4, 5, 6, 7, 1907.—*American Medical Journalist, New York.*

By order of The Octopus, Simmons, Lord High Chief Executioner.

THE AMERICAN MEDICAL JOURNALIST for March is a red-hot number, sparkling with good things, and is most intensely interesting reading—editorials especially. It is commended to the self-righteous—the "unco-gude" of the Octopus persuasion.

---

THE SIXTEENTH INTERNATIONAL MEDICAL CONGRESS will meet at Buda Pest, Hungary, August 29 to September 4, 1907.

---

THE FOLLOWING is from one of the independent medical publishers who has the courage to resent, as we do, the high-handed and outrageous course of the Octopus and its sycophants. The independent medical press should organize resistance to the Medical Journal Trust:

"Your March issue came to hand a couple of days ago and your leading editorial fully indicated that, like myself, you were no more able to keep out of the fray—under existing intolerable conditions—than is the swimsome youngster able to keep out of the pond at the burst of spring. If medical publishers all over the country would only make up their minds to lock horns with the "Machine" in one unanimous bunch, they would make such an impression upon the Octopus as might possibly astonish themselves at the effects of their own strength. Where, however, only here and there a publisher gets busy, he is comfortably picked off as a single spy by the "Machine," when a battalion would be more than it could handle. It has been a matter of standing wonder to me that medical publishers at large have not recognized this tactical condition and acted accordingly. I also wonder, whether or not, they will ever get down to it. What do you think?

---

DIED ON THE CALENDAR. The 30th Texas Legislature adjourned April 12 and was immediately reassembled by the Governor to consider platform demands only.

The Castration for Rape bill, the Tuberculosis Sanitarium bill, the State Board of Health bill, all died, not reached. The joint conference cut out the little appropriation to make effective the vital statistics law, and Brumby has about two cart loads of reports on hand and no way to use them. Comment is unnecessary. I can't do justice to the subject.

---

How dear to our heart is the cash on subscription,
When the generous subscriber presents it to view;
But the man who won't pay we refrain from description,
For perhaps, gentle reader, that man may be you.

—*Ex.*

---

PROFESSOR FITCH: Our handsome and distinguished friend, Dr. Wm. E. Fitch, of Gaillard's Southern Medicine, New York, has been elected Demonstrator of Anatomy, Medical Department, Fordham University, New York.

---

THE BILLS THAT GOT THROUGH are the Pure Food bill, the Mixed Board monstrosity, the Anatomical bill, $75,000 for additions to Southwestern Insane Asylum, San Antonio, and an asylum for idiots, $25,000, to be annexed to the Austin State Lunatic Asylum. No action was taken on the Federal Quarantine law requiring States to turn over international quarantine to the United States government.

---

THE LATEST.—April 17th. The Governor today signed the Mixed Board Bill. The "Red Back" is the first to publish the news and the bill.

---

THE DR. NATHAN SMITH DAVIS MEMORIAL. — I desire to call the attention of the members of the medical profession of Texas to the fact that a committee has been appointed by the American Medical Association to collect a fund to be devoted to establishing a suitable memorial to the late Dr. N. S. Davis, one of the founders of the American Medical Association, one of the authors of the original code of ethics, founder and first editor of the *Association Journal.* It seems appropriate that the memory of a man who did so much for medical organization and for the progress of American medicine should be perpetuated in a suitable way.

The undersigned having been appointed a member of the committee from Texas, with authority to add the names of other members to the Texas division, begs leave to state that he has requested the Councilors of Texas to serve on this committee, believing it most appropriate to form the committee of these gentlemen with authority to aid in collecting for this fund. A number of county societes have already contributed. It is hoped that good progress can be made in this worthy cause in time for a creditable report to be made to the American Medical Association at its annual meeting on June 4th, when it is desired to formulate plans for beginning the work. This is a subject that appeals to the pride and pleasure of members of the profession. Any amount that any member feels disposed to contribute will be received with thanks, and no member need consider himself debarred from the privilege of contributing because he does not feel able to give a large sum, as it matters not how small the contribution. It is much more desirable to have a large number identified in establishing this memorial than a few.

Hoping the profession will be interested in this movement, I am, very respectfully, J. T. WILSON.

---

"NO NOSTRUMS BEAR OUR LABEL" is a text that hits the nail on the head. The hue and cry anent nostrums does not in the least disturb the Baltimorean equanimity of one of our old advertisers for the good and sufficient reason that they "haint got some."

When the roll of the ethical purveyors to the medical profession is called, these old friends will say "here" in no uncertain tones.

The sermonette on "Ethical Products vs. Nostrums," on our front cover this month, should not escape the eye of any member of the "Red Back" family. What we all want is more light, the kind of light that really illumines, not the kind that blinds. Selah.

---

JOURNAL CONSOLIDATION—Dr. Clarence Martin, of the *Medical Era,* has purchased the *Medical Mirror* and merged it into the *Medical Era.* The combine will be known as the *Medical Era.*

---

NEW YORK, March 13, 1907.

*Texas Medical Journal, Austin, Texas.*

GENTLEMEN:—On the morning of March 4th we were visited by fire, which practically destroyed the manufacturing end of our business. We had, however, a duplicate plant in storage and are pleased to state that, after four days and nights of continuous work, we were again turning out Glyco-Thymoline. We regard this as a record. Yours very truly,

KRESS & OWEN COMPANY.

